# Unintentional Child and Adolescent Drowning Mortality from 2000 to 2013 in 21 Countries: Analysis of the WHO Mortality Database

**DOI:** 10.3390/ijerph14080875

**Published:** 2017-08-04

**Authors:** Yue Wu, Yun Huang, David C. Schwebel, Guoqing Hu

**Affiliations:** 1 Department of Environmental and Occupational Health, Xiangya School of Public Health, Central South University, 110 Xiangya Road, Changsha 410078, China; wuyue7802@csu.edu.cn (Y.W.); 803110@csu.edu.cn (Y.H.); 2 Department of Psychology, University of Alabama at Birmingham, Birmingham, AL 35294, USA; schwebel@uab.edu; 3 Department of Epidemiology and Health Statistics, Xiangya School of Public Health, Central South University, 110 Xiangya Road, Changsha 410078, China

**Keywords:** drowning, child, mortality, trend

## Abstract

Limited research considers change over time for drowning mortality among individuals under 20 years of age, or the sub-cause (method) of those drownings. We assessed changes in under-20 drowning mortality from 2000 to 2013 among 21 countries. Age-standardized drowning mortality data were obtained through the World Health Organization (WHO) Mortality Database. Twenty of the 21 included countries experienced a reduction in under-20 drowning mortality rate between 2000 and 2013, with decreases ranging from −80 to −13%. Detailed analysis by drowning method presented large variations in the cause of drowning across countries. Data were missing due to unspecified methods in some countries but, when known, drowning in natural bodies of water was the primary cause of child and adolescent drowning in Poland (56–92%), Cuba (53–81%), Venezuela (43–56%), and Japan (39–60%), while drowning in swimming pools and bathtubs was common in the United States (26–37%) and Japan (28–39%), respectively. We recommend efforts to raise the quality of drowning death reporting systems and discuss prevention strategies that may reduce child and adolescent drowning risk, both in individual countries and globally.

## 1. Introduction

Children and adolescents are vulnerable for drowning because they are mobile, curious, and like to explore their environment [1], and also because they lack adult levels of risk consciousness and water-safety knowledge [2]. According to the latest Global Burden of Disease (GBD) estimates, 127,577 children and youth under the age of 20 years died from drowning in 2015 in the world, constituting over 39.4% of all-age drowning deaths [3].

Assessment of the epidemiological characteristics and monitoring of trends in drowning at the national level is critical to understand the mechanisms of drowning and develop feasible solutions to reduce risk. However, only a few studies report recent child drowning mortality at the national level for multiple countries. GBD 2015 estimates are helpful to the field, as they provide national estimates of child and adolescent drowning rates for 195 countries, and subnational estimate for seven large countries [4]. GBD data are, however, limited in their scope. They combine all methods of drowning into a single category and thus do not offer data concerning the frequency of drowning from different methods or causes.

Existing research has quantified some aspects of drowning rates around the world. Huang et al. [5], for example, used GBD 2013 data and reported large decreases in global under-5 years of age drowning mortality between 1990 and 2013, including a greater drop in drowning mortality in developing countries compared to developed countries. Other reports have focused on changes in drowning rates in single countries or regions, such as manuscripts by Dai et al. in Georgia [6]; Anary et al. in Mazandaran Province, Iran [7]; Tyebally and Ang in Singapore [8]; and Ahlm et al. in Sweden [9]. A few studies have extended broad information on drowning rates to examine rates by method and cause. One recent paper, for example, used the combined mortality data for the latest available three years (2004–2011) in the WHO Health Statistics and Information Services to examine differences in unintentional drowning mortality by age and body of water across 60 countries, revealing huge variations in age-adjusted mortality rate, from 0.12 in Turkey to 9.19 per 100,000 persons in Guyana; the authors ascribed the large country variations to be resulting from differences in coding practice for drownings [10]. A report in the mid-1990s by the Wet International Collaborative Effort (ICE) Collaborative group revealed great variations in reported drowning with undetermined intent across countries [11]. Almost 40% of drowning deaths in England and Wales were coded as having undetermined intent, for example, but only 5% in the United States and New Zealand and 1% in Israel were coded as having undetermined intent. Unfortunately, no published study has examined this issue using recently released drowning mortality data.

The present study was designed to study these issues in greater detail. We considered three specific research questions: (a) Did the included countries experience similar change in child and adolescent drowning mortality between 2000 and 2013? (b) Did the countries follow the same trends in the method of drowning across the study time period? and (c) Did the countries have the same portion of child and adolescent drowning with unspecified methods, and did the portion change between 2000–2013 for each of the countries?

## 2. Methods

### 2.1. Data Source

Drowning mortality data were obtained from the WHO Mortality Database [12], which provides raw death registration data over many years for about 150 countries and regions. Twenty-one eligible countries were included in our study, based on their meeting the following four criteria: (1) death registration system covered ≥70% of the country population; (2) data were available for 10 or more years between 2000 and 2013; (3) ≥100 drowning deaths were reported for people under the age 20 for each year with reported data; and (4) the 10th International Classification of Disease (ICD-10) was used to code deaths for data analysis. Coverage data of death registration systems were from the estimates of the Institute for Health Metrics and Evaluation (IHME) [13,14,15].

### 2.2. Choice of Age Group

The Convention on the Rights of the Child [16] defines children as persons under 18 years of age. The WHO Mortality Database, however, does not provide mortality data for individuals under the age of 18 years as a single group, nor does it provide data for each age in years that could be used to calculate the mortality of an under-18 years age group. Instead, it presents mortality data for the under-20 years age group. Developmentally, 18- and 19-year-olds are similar in terms of their cognitive and social functioning to adolescents who are age 16- and 17-years old [17], so given developmental theory and the availability of data in the WHO database, our analyses focused on the under-20 years age group. We use the term, “child and adolescent” in this manuscript to represent those individuals under 20 years of age.

### 2.3. Classification of Drowning

Unintentional drowning deaths were identified using ICD-10 codes “W65–W74”. Based on preliminary analysis (not shown here), we grouped unintentional drowning into five sub-causes by method of drowning: drowning in a bathtub (W65–W66); drowning in a swimming pool (W67–W68), drowning in natural bodies of water (W69–W70), other specified drowning (W73), and unspecified drowning (W74).

### 2.4. Classification of Countries

We adopted the classification of the World Bank in 2013 to classify countries into low- and middle-income countries (LMICs) and high-income countries (HICs) [18].

### 2.5. Statistical Analysis

Three countries had missing data in certain years (Cuba, 2000; Philippines, 2004, 2005, 2012, 2013; Thailand, 2001). In these cases, we imputed the missing data using the methods recommended by the WHO [14]. Specifically, missing values that appeared at the starting or ending years of 2000 or 2013 were extrapolated by replacing them with the mean death rate of the first or last three years. Missing values that appeared in the middle of the time span from 2000 to 2013 were interpolated by replacing them with the mean death rate of all available data in a seven-year window (three years on either side around the year with missing values) [14].

The portion of drowning attributed to unspecified causes (method) was used to measure the specificity of the sub-cause (method) for drowning and the quality of the data available. A large portion of unspecified sub-causes (method) for drowning generally indicates low sub-cause (method) specificity.

We calculated the age-standardized mortality rate from 2000 to 2013 using the direct standardization method. The new WHO world standard population (WHO millennium) was used as the reference population to perform the direct standardization method [19]. Subgroup analyses by age group were not conducted due to insufficient numbers of drowning deaths, which may cause unstable mortality rates. The percent change in rate was calculated as “(mortality in 2013−mortality 2000)/mortality rate in 2000 × 100%”. Pearson chi-square test was used to compare differences in mortality rates between 2000 and 2013.

### 2.6. Ethics Statement

This study was based on open access data and therefore was exempted from ethics review. This research plan was approved by the Medical Ethics Committee of Central South University (No. XYGW-2016-25).

## 3. Results

Of the 150 countries and territories included in the WHO Mortality Database, 89 had drowning death data available for 10 or more years between 2000 and 2013. Of those 89 countries and regions, five (Dominican Republic, Kuwait, Nicaragua, Peru, Qatar) were excluded because their death registration system covered less than 70% of the country’s population, and another 63 were excluded because the number of annual drowning deaths observed was less than 100, making subgroup analysis by method of drowning invalid. This left 21 countries that met the inclusion criteria for our analysis; 14 LMICs and 7 HICs (Table 1). Between 2000 and 2013, a total of 107,350 and 25,741 under-20 drowning deaths occurred in those 14 LMICs and 7 HICs, respectively.

Under-20 drowning mortality rate varied greatly across countries and over time between 2000 and 2013 (Table 1). In general, HICs had lower drowning mortality rates in the study time period compared to LMICs. Of the 21 included countries, the highest mortality rate was in Kyrgyzstan (8.10 per 100,000 persons) in 2000, while the lowest mortality rate occurred in South Africa (0.33 per 100,000 persons) in the same year, a 24-fold difference between the two countries. In 2013, the largest drowning mortality gap occurred between Thailand (6.14 per 100,000 persons) and Germany (0.34 per 100,000 persons). Drowning mortality rates of the 14 LMICs included in this study, which ranged from 1.19 to 6.14 per 100,000 persons in 2013 (mean = 2.76), were consistently higher than those in the 7 HICs (which ranged from 0.34 to 1.08; mean = 0.79).

Twenty of the 21 countries experienced a reduction in their drowning mortality rate between 2000 and 2013 (ranging from −80 to −13%). South Africa, which witnessed a 924% increase, was the exception (Table 1). HICs generally experienced a larger decrease in their under-20 drowning mortality rate compared to LMICs.

**Figure 1:**
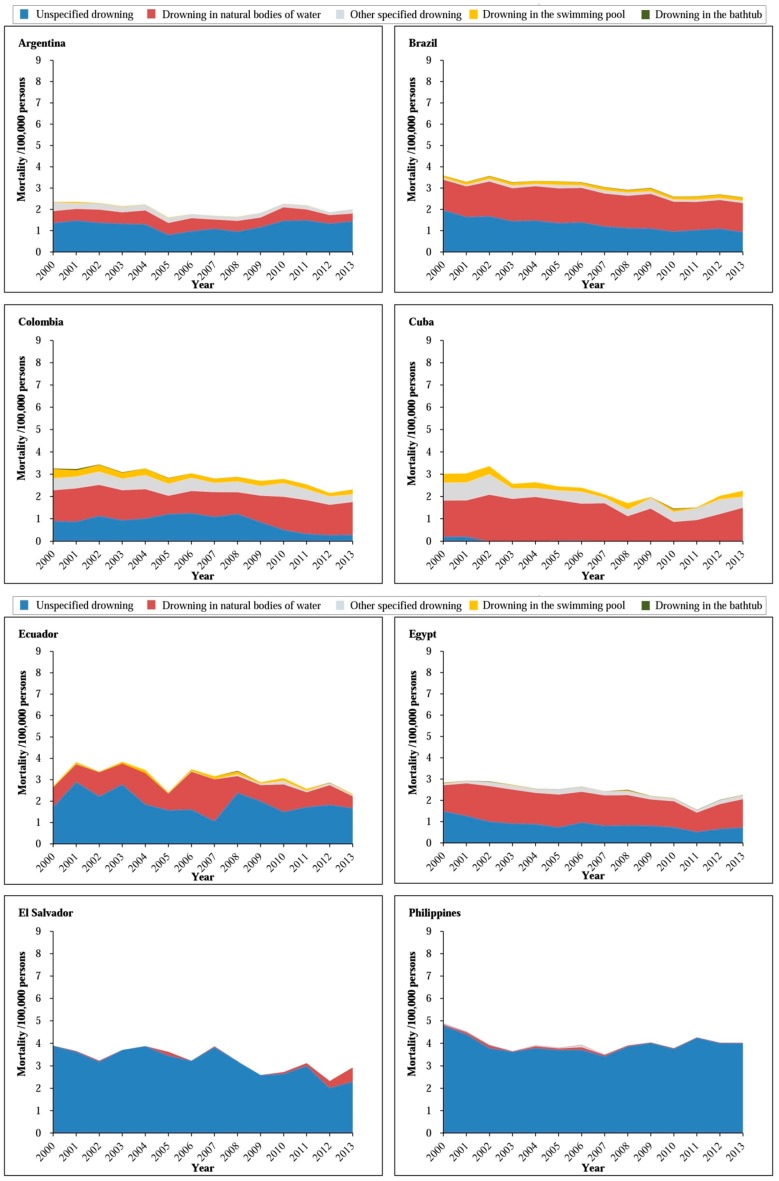

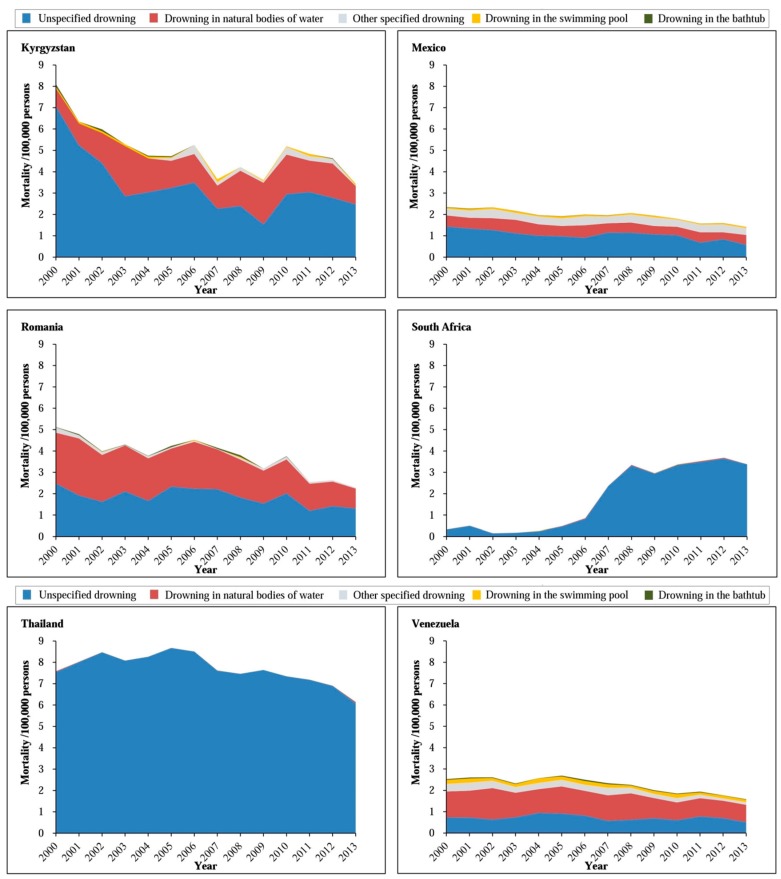
Trends in the method of under-20 unintentional drowning rate in 14 low- and middle-income countries, 2000–2013.

**Figure 2:**
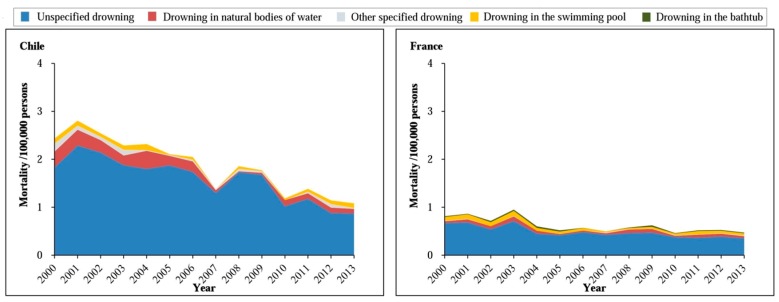

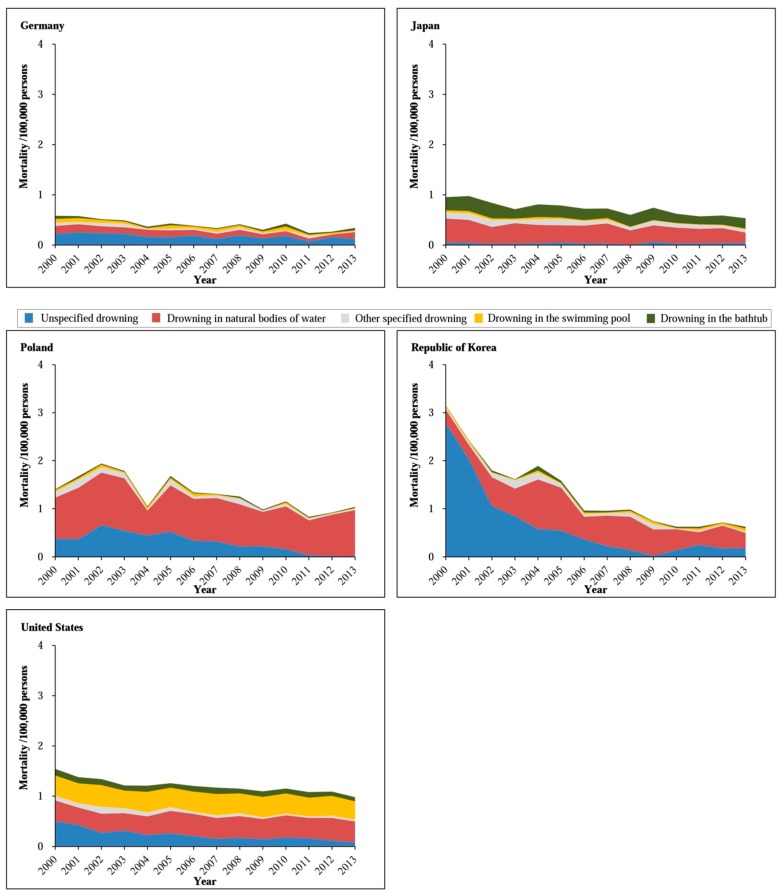
Trends in the method of under-20 unintentional drowning rate in seven high-income countries, 2000–2013.

A detailed analysis of the method of drowning in each countries revealed that drowning in natural waters was the most common method of under-20 drowning in Poland (56–92%), Cuba (53–81%), Venezuela (43–56%), and Japan (39–60%). Drowning in swimming pools was comparatively common in United States (26–37%) and drowning in bathtubs frequently occurred in Japan (28–39%) (Figure 1 and Figure 2).

**Figure 3:**
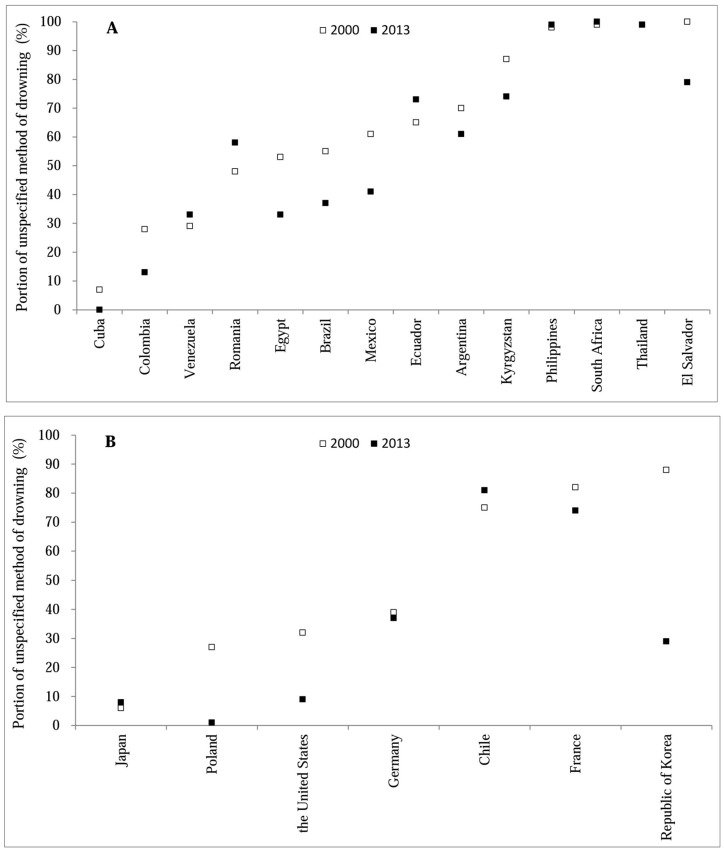
Portion of drowning with unspecified methods in 21 countries, 2000–2013: (**A**) Low- and middle-income countries; (**B**) High-income countries.

Different portions of unspecified methods and inconsistent patterns of change between 2000 and 2013 across countries appear in Figure 3. During the time period between 2000 and 2013, the portion of drowning with unspecified methods was over 70% in five countries (Thailand, 99–100%; South Africa, 92–100%; El Salvador; 87–100%; Chile, 75–95%; France, 74–85%). In contrast, it was less than 20% in Cuba and Japan. Between 2000 and 2013, 13 of the 21 countries experienced reductions in the portion of drowning with unspecified methods. The largest reductions were seen in Cuba (100%), Poland (96%), United States (72%), Republic of Korea (67%), and Colombia (54%).

## 4. Discussion

To summarize, this study yielded four important findings: (1) the unintentional under-20 drowning mortality rate decreased between 2000 and 2013 in 20 of the 21 included countries; (2) LMICs generally had higher mortality rates and experienced smaller reductions in their under-20 drowning mortality rate over the study time period compared to HICs; (3) drowning in natural bodies of water was the primary method of under-20 drowning in Poland, Cuba, Venezuela, and Japan, while drowning in swimming pools and bathtubs was common in the United States and Japan, respectively; and (4) specificity in drowning method reporting greatly varied across countries.

Drowning is a preventable public health problem. The general decrease in drowning mortality between 2000 and 2013 likely reflects the effects of global prevention efforts. A framework, initially developed by the International Life Saving Federation (ILSF) in 2008, was updated to a third version in 2015 that includes a description of how the ILSF framework has been applied to assist nations and organizations to provide the best response to drowning reduction [20]. In the last three decades, a number of effective child drowning prevention strategies have been developed and implemented; these include removing (or covering) water hazards, requiring isolation fencing (four-sided) around swimming pools, wearing properly-fitted personal flotation devices (PFDs), and ensuring immediate resuscitation [21]. Such strategies have been implemented across many countries. For example, PFD loaner programs have been placed across the United States at beaches and boat ramps to promote PFDs use, usually at no charge [22]. Community education and lifeguard training programs have been carried out in a number of LMICs, including Brazil and Thailand [20].

We found large child and adolescent drowning mortality differences among individual countries, both between LMICs and HICs and also across countries within those two groupings. Higher drowning mortality risks in LMICs are likely the result of a variety of factors, including lack of knowledge and safety awareness about water safety; limited swimming ability among children and adult supervisors; inadequate application of interventions (e.g., using barriers around swimming pools and open bodies of water); lesser adult (parent and professional lifeguard) supervision near water; more frequent interaction with water for daily living (washing, drinking, bathing); and for adolescents especially, swimming during or after drinking or drug use and rough or risky play in and near water with peers [21,23,24,25,26].

Apart from inconsistent drowning mortality changes across countries, the 924% increase in child and adolescent drowning deaths between 2000 and 2013 in South Africa is surprising. A similar abnormal increase for drowning mortality was reported in Mthatha, South Africa between 1993 and 2004 (an increase from 2.7 to 12.0 per 100,000 persons) [27]. These unusual increases may be a result of changes in data reporting in South Africa rather than actual changes in drowning deaths. Further analysis (not shown here) indicates that drowning deaths with undetermined intent decreased from 727 in 2000 to 540 in 2006 and after then remained close to 0 for South Africa, accompanied with a jump in unintentional drowning deaths since 2006. We know, for example, that there has been improved use of valid methods such as verbal autopsy interviews to assign the cause of death in South Africa [28]. There also has been substantial improvement in the completeness of death registration records, especially in rural areas of the country (from 79.8% in 2000–2004 to 93.4% in 2010–2014) [29].

A detailed subgroup analysis concerning the method of child drowning revealed large variations in the cause of drowning across countries. Drowning in swimming pools was common in the United States (26–37%), a result that matches previous reports [30]. Drowning in the bathtub was common in Japan (28–39%), which also accords with previous studies [31,32]. In most of the rest of the world, drowning in natural bodies of water was most common (e.g., in Poland [33]). In general, these patterns may reflect exposure opportunity. Poland is a country with much natural water [34] and children spend much time near those bodies of water. In Japan, it is common practice to leave water sitting in bathtubs for long periods of time so that the water can be re-used for bathing [32]. The United States has many swimming pools, including a large number of pools in backyards that are not monitored by lifeguards [35]. For these reasons, child drowning prevention priorities must be tailored based on geographical, financial, and cultural conditions that reflect significant drowning risks in each country.

We considered the portion of unspecified cause of child drowning death as a measure of the reporting quality in each country. Although the quality of reporting on method specificity for drowning improved in 13 of the 21 countries, only two LMICs (Cuba, Colombia) and three HICs (Japan, Poland, United States) had less than 20% unspecified drowning deaths in 2013. To maximize the value of surveillance data, further efforts should be made to raise the specificity and therefore quality of death registration in countries lacking high-quality mortality data.

Our findings have several implications. First, they indicate incremental success in preventing under-20 drowning mortality in 20 of the 21 countries we included. Nevertheless, drowning remains a major cause of child and adolescent death throughout the world. According to the GBD 2015 estimates, 127,501 under-20 children and adolescents died from drowning in the world [3], making drowning the second leading cause of under-20 injury mortality globally. Furthermore, the burden from child and adolescent drowning affects LMICs at a highly disproportionate rate, with 97.8% of global child deaths from drowning occurring in LMICs even though only 88.7% of the global child population lives in those countries. These data underscore the urgency of the development and implementation of globally coordinated, theory-based, and effective drowning prevention efforts.

A second implication of our results is the need to improve data specificity in many countries. Some nations we studied—including Thailand, South Africa, Philippines, and Chile—recorded over 80% of child drowning deaths as a result of unspecified causes. Improved specification of drowning methods is crucial for identifying major sub-causes of drowning and then tailoring intervention priorities to match causal factors.

Our analysis has two clear strengths. First, it offers detailed epidemiological evidence concerning drowning of children and adolescents under the age of 20 years, including epidemiological data on the method of drowning. These results provide valuable information for implementing drowning prevention policies and developing prevention strategies and priorities. Second, our analysis has methodological strengths, including the exclusion of countries with poor surveillance system coverage; with an absence of mortality data for four or more years; and with under-20 drowning mortality with fewer than 100 deaths, which would limit the estimation of a stable subgroup mortality. These methodological strengths ensure that our results are based on the impact of adequate data and stable mortality estimates.

Our analysis is limited somewhat by data availability. Some countries with substantial numbers of child drowning deaths and/or high drowning mortality rates that are covered by other non-open access datasets were excluded because they did not report data to the WHO mortality database, including China, India, and Bangladesh. Second, we excluded countries with small populations because subgroup mortality rates may have been unstable due to small numerators. Finally, we excluded countries with low completeness and/or with low death registration system coverage according to WHO criteria [14,15]. These countries represent a non-random set of exclusions.

## 5. Conclusions

Twenty of 21 included countries witnessed a moderate or minor decrease in under-20 drowning mortality between 2000 and 2013. We found substantial variations in drowning mortality and mortality change between 2000 and 2013, both between HICs and LMICs, and across countries within those groupings. We recommend more efforts be taken to reduce child drowning globally, and especially in LMICs, with attention paid to the highest risks in each country. For example, non-governmental organizations such as WHO and UNICEF could promote international coordination between developed and developing countries to enhance the drowning prevention capacity in developing countries. We also recommend efforts be taken to improve the quality of drowning death reporting at global and national levels, including completeness, cause specificity, accuracy of cause classification, timeliness, and open access of drowning mortality data.

## Figures and Tables

**Table 1:** Unintentional child and adolescent drowning deaths and age-adjusted mortality per 100,000 persons for 21 countries, 2000–2013.

Country	2000		2013		Percent Change in Rate
	Deaths	Mortality Rate	Deaths	Mortality Rate	
**Low- and middle-income countries**					
Argentina	320	2.19	184	1.19	−46 **
Brazil	2657	3.59	1740	2.57	−28 **
Colombia	587	3.25	390	2.31	−29 **
Cuba	100	3.01	62	2.25	−25
Ecuador	165	2.69	153	2.33	−13
Egypt	958	3.89	850	2.93	−25 **
El Salvador	102	3.40	75	2.34	−31
Philippines	1893	4.88	1684	4.03	−17 **
Kyrgyzstan	196	8.10	98	3.43	−58 **
Mexico	1137	2.35	711	1.42	−40 **
Romania	327	5.12	99	2.28	−55 **
South Africa	74	0.33	762	3.38	924 **
Thailand	1606	7.60	1066	6.14	−19 **
Venezuela	286	2.53	193	1.58	−38 **
**High-income countries**					
Chile	136	2.43	58	1.08	−56 **
France	135	0.81	81	0.47	−42 **
Germany	105	0.58	54	0.34	−41 **
Japan	262	0.95	127	0.53	−44 **
Poland	163	1.41	87	1.04	−26 *
Republic of Korea	447	3.15	73	0.62	−80 **
United States	1314	1.54	866	0.98	−36 **

Mortality rate was standardized using the new World Health Organization (WHO) world standard population (WHO millennium) as a reference. The percent change in rate was calculated as “(mortality in 2013−mortality 2000)/mortality rate in 2000 × 100%”. *: *p* < 0.05; **: *p* < 0.01.
